# Nail Apparatus Melanoma: Current Management and Future Perspectives

**DOI:** 10.3390/jcm12062203

**Published:** 2023-03-12

**Authors:** Takamichi Ito, Hiroki Hashimoto, Yumiko Kaku-Ito, Yuka Tanaka, Takeshi Nakahara

**Affiliations:** Department of Dermatology, Graduate School of Medical Sciences, Kyushu University, Fukuoka 812-8582, Japan

**Keywords:** nail melanoma, nail unit melanoma, acral melanoma, acral lentiginous melanoma, antibody-drug conjugate, CAR-T, tebentafusp

## Abstract

Nail apparatus melanoma (NAM) is a rare type of cutaneous melanoma that belongs to the acral melanoma subtype. NAM is managed principally in accordance with the general treatment for cutaneous melanoma, but there is scarce evidence in support of this in the literature. Acral melanoma is genetically different from non-acral cutaneous melanoma, while recently accumulated data suggest that NAM also has a different genetic background from acral melanoma. In this review, we focus on recent advances in the management of NAM. Localized NAM should be surgically removed; amputation of the digit and digit-preserving surgery have been reported. Sentinel lymph node biopsy can be considered for invasive NAM for the purpose of accurate staging. However, it is yet to be clarified whether patients with metastatic sentinel lymph nodes can be safely spared completion lymph node dissection. Similar to cutaneous melanoma, immune checkpoint inhibitors and BRAF/MEK inhibitors are used as the first-line treatment for metastatic NAM, but data on the efficacy of these therapies remain scarce. The therapeutic effects of immune checkpoint inhibitors could be lower for NAM than for cutaneous melanoma. This review highlights the urgent need to accumulate data to better define the optimal management of this rare melanoma.

## 1. Introduction

Nail apparatus melanoma (NAM) is a distinct type of cutaneous melanoma occurring in the nail apparatus of the hand and foot, which belongs to the acral melanoma subgroup. Although acral melanoma has similar incidences across different ethnic groups [1,2], it accounts for a higher proportion of melanoma cases in darker-skinned individuals since non-acral melanomas are less common in people of color [3,4]. Complete removal of the tumor at an early stage is curative, while invasive NAM increases the risk of lymph node or distant metastasis. Early detection and prompt therapeutic intervention are therefore important. However, there are several mimics of NAM and the diagnosis may sometimes be delayed. Systemic therapies are required for unresectable or metastatic NAM. In this review article, we present an overview of recent advances in the treatment of NAM and discuss the management of this condition.

## 2. Materials and Methods

We have conducted a narrative review by browsing the PubMed databases with the keywords “acral”, “nail” and “melanoma”. We have selected original articles, case reports, and review articles written in English. Several reports were also adopted from the References in the selected articles.

## 3. Epidemiology

NAM, comprising 0.7–3.5% of all cutaneous melanomas [5], accounts for 0.18–2.8% of melanomas in Europeans, 10–23% in Asians, and 25% in African–Americans [6]. NAM typically occurs in older people (mean age at diagnosis of 60–70 years old) [6], but on very rare occasions, pediatric cases have been reported [7,8]. The most commonly affected anatomical locations are the thumbnail and big toenail [9,10]. Common risk factors for cutaneous melanoma, such as fair skin, sun exposure, and a family history of melanoma, do not apply to NAM [11,12]. Instead, trauma, chronic inflammation, and mechanical stress, which are thought to contribute to acral melanoma development [13,14,15,16], may also play some roles in NAM development [17]. A recent report comparing NAM with non-NAM acral melanoma (54 NAMs and 78 non-NAM acral melanomas) reported that patients with NAM were younger at diagnosis, had a higher prevalence of primary melanoma on the hand, and had more frequent reports of previous trauma at the tumor site [18]. Like other types of melanomas, most NAM cases occur in the epidermis (nail bed epithelium) as an in situ tumor. Regarding the prognostic factors, a large cohort study (2050 acral melanomas) revealed that acral melanoma shares the same prognostic factors (age, ulceration, tumor thickness, and tumor spread) as other types of melanomas [19], and NAM presumably has similar prognostic factors [20,21]. A large retrospective study in China (1157 acral melanomas, including 270 NAMs) reported that NAM and palmar acral melanoma had a better prognosis than plantar acral melanoma [21].

## 4. Diagnosis

### 4.1. Clinical Findings

**Figure 1:**
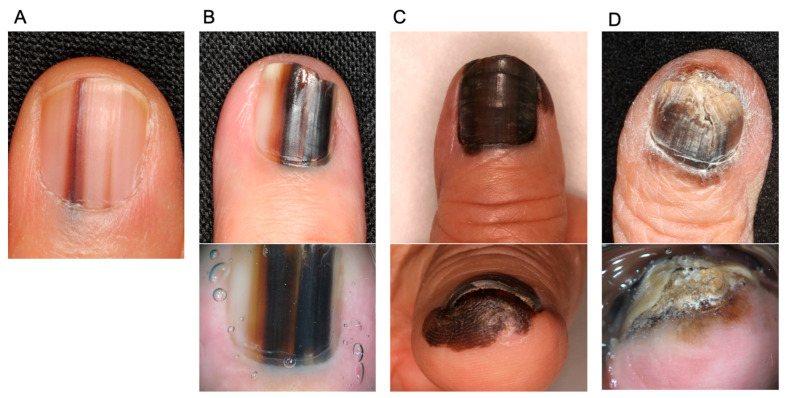
Representative images of nail apparatus melanoma and dermoscopy (lower images). (**A**): An early lesion of nail apparatus melanoma. Both of the streaks taper toward the distal end of the nail. (**B**): An invasive nail apparatus melanoma. The color is highly irregular. (**C**): An invasive nail apparatus melanoma. The entire thumbnail is deeply pigmented, accompanied by Hutchinson’s sign. (**D**): An invasive toenail apparatus melanoma. Hutchinson’s sign and nail destruction are evident.

NAM at an early stage generally presents longitudinal melanonychia, which is irregular in width and color. In contrast, benign melanocytic nevus, which is a major differential diagnosis, is characterized by brown or black pigmented streaks extending from the proximal nail fold to the distal end of the nail plate. Typical melanocytic nevus is regular in color with a stable width throughout the nail. Extended pigmentation to the surrounding skin (Hutchinson’s sign), widening of melanonychia toward the proximal nail, destruction of the nail plate, bleeding, localization to one nail, and rapid enlargement and darkening during adulthood are all signs suggestive of NAM. However, information beyond the clinical appearance, such as age, history, and involvement of other nails, is important and should be considered when deciding on the appropriate treatment [9,22,23,24,25,26]. Representative images of NAMs are shown in Figure 1.

Like melanomas at other sites, dermoscopy improves the diagnostic accuracy of NAM [22,27,28]. Pigmentation of NAM typically involves more than two-thirds of the nail plate, while that of benign melanonychia shows an involvement of less than one-third. Irregularly pigmented streaks, Hutchinson’s and micro-Hutchinson’s signs, and nail destruction are other dermoscopic clues of NAM. Notably, these clues are more common in benign melanonychia of children than that of adults [22,29]. Congenital nevi of the nail matrix may mimic melanoma, but NAM in prepubescent children is exceedingly rare. Therefore, a nail matrix biopsy in children is considered only in exceptional cases [22], since it may permanently deform the nail. A close follow-up is a judicious approach for clinically ambiguous cases.

**Figure 2:**
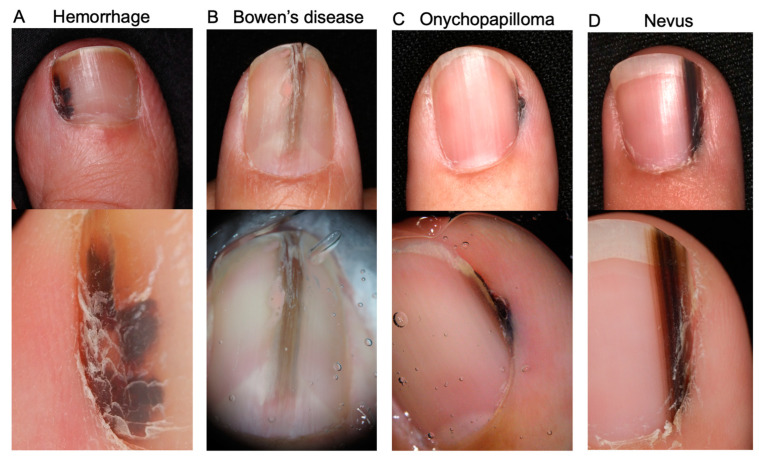
Representative images of differential diagnoses of nail apparatus melanoma together with their dermoscopy images (lower images). (**A**): Subungual hemorrhage. Reddish black macules are evident in dermoscopy. (**B**): Subungual Bowen’s disease. (**A**) A slightly elevated brown longitudinal streak is observed in the middle of the nail bed. Nail deformity is evident. (**C**): Onychopapilloma. An irregular melanonychia in the lateral nail fold. (**D**): Melanocytic nevus. The melanonychia consists of regularly arranged brown to black streaks. No nail deformity or Hutchinson’s sign is observed.

Clinical differential diagnoses of NAM include a wide range of benign and malignant conditions, namely, subungual hemorrhage, fungal melanonychia, onychopapilloma, onychomatricoma, Bowen’s disease/squamous cell carcinoma in the nail apparatus, and benign melanocytic macules [Figure 2] [30,31,32]. A definitive diagnosis is made by histopathological analysis. For biopsy, the nail matrix area should be sampled since NAM typically arises from melanocytes in the nail matrix [23]. It is generally easy to distinguish NAM from the above-mentioned differential diagnoses, with the exception of melanocytic nevus, since these conditions have distinct histopathological features (e.g., atypical keratinocyte proliferation in Bowen’s disease). The differentiation between NAM and benign melanocytic macules is discussed in Section 4.3. Histopathological findings section.

### 4.2. Treatment of Obtained Samples

Particular attention should be paid to the fixation and decalcification of the obtained tissue so as to preserve the tissue quality at a level suitable for genetic analysis (*BRAF* mutations, etc.). For example, a fresh surgical tissue sample should be quickly immersed in formalin (ideally within 30 min) but not immersed for a long time (6–72 h).

Specific decalcifying agents should be selected for preserving tumor DNA/RNA. For amputated finger samples, bone samples can be separated before (or after) formalin fixation to minimize the decalcification process, although caution should be taken so as not to damage the tumor in order to accurately measure the tumor thickness.

The use of tissue softeners may be an aid in sectioning nail tissue [33,34].

### 4.3. Histopathological Findings

Histopathological diagnostic criteria of NAM are similar to those of other types of melanomas. The diagnosis is based on multiple criteria, including architectural and cytological features. Architectural features suggestive of melanoma include asymmetry, poor circumscription, consumption of the epidermis, haphazard interval, and irregular arrangement. Cytological features include nuclear pleomorphism, nuclear variability, and atypical mitoses. Histopathologically, most NAM cases are of the acral lentiginous type [35], which is characterized by the proliferation of atypical melanocytes along the basal layer of the epidermis [36]. At a very early stage, there may be only scattered lentiginous, atypical melanocytes. Lymphocytic infiltrates, pagetoid scatter, and cytological atypia with mitoses then become evident with time [36].

Compared with NAM, nevus in the nail apparatus is relatively small and circumscribed. Although nevus in the nail matrix belongs to the category of “special-site” nevi that may show atypical histopathological features, pagetoid scatter and bridging between rete ridges tend to be sparse. The diagnosis of NAM at an early stage is sometimes challenging since the histopathological changes are subtle [23]. To identify melanocytes and melanoma cells in the nail matrix, immunochemical staining using Melan-A, HMB45, S-100, SOX-10, and MITF is useful [22].

To date, no definitive marker that can distinguish early melanoma from benign melanocytic lesions has been established. A recent study found that p16, HMB45, and Ki-67/Melan-A staining did not distinguish benign activated melanocytes from nail apparatus melanoma in situ [37]. Recently, PRAME, a cancer testis antigen, has attracted attention as a useful marker in the differential diagnosis between benign and malignant melanocytic lesions [38,39,40]. An immunohistochemical study investigating 22 NAMs, 20 non-NAM acral melanomas, and 14 benign acral nevi reported that 73% of NAMs, 95% of non-NAM acral melanomas were positive for PRAME at least in part, whereas only one (7%) acral nevus exhibited PRAME expression [38]. In a study on 127 acral melanocytic lesions (including 20 nail melanocytic lesions), a PRAME expression score calculated by percentage positivity and expression intensity demonstrated good sensitivity and specificity in the diagnosis of acral melanocytic lesions [39]. However, the authors also identified a subset of challenging cases such as acral Spitz nevi, in situ melanomas, and small, thin, invasive melanomas [39]. In contrast, another study comparing 14 benign subungual melanocytic proliferations and 13 in situ NAMs showed that PRAME nuclear immunostaining (cutoff of 10%) exhibited good overall discrimination between benign melanocytic proliferation and NAM in situ [40]. Collectively, although not perfect, PRAME will aid for the diagnosis of NAM under the appropriate cutoff or scoring. Fluorescence in situ hybridization (FISH) can provide additional information [41,42,43], but it may not be routinely available.

### 4.4. Genetic Findings

Cutaneous melanoma is a tumor rich in genetic alterations [44]. Most such cases have coding and noncoding mutations primarily driven by exposure to ultraviolet (UV) radiation, whereas acral and mucosal melanomas present with fewer mutations of the UV signature [45,46]. Interestingly, melanomas at acral sites show distinct patterns based on the site and relative sun exposure, and accumulating evidence suggests that NAM is distinct from acral melanoma [19,47,48,49]. For instance, a recent study showed evidence of a UV signature in 85.7% (6/7) of NAMs on the fingernails and 33.3% (5/15) of them on the toenails [49]. Mutations found in melanomas on the dorsal hand and foot were reported to be similar to those in melanomas at other sites of intermittently sun-damaged skin, whereas subungual and interdigital melanomas (*n* = 13) exhibited diverse mutations in *PIK3CA*, *STK11*, *EGFR*, *FGFR3*, and *PTPN11* [48]. Copy number aberrations were common (8/12, 67%), particularly in *CDK4* and *CCND1* [48]. One study comparing NAM (*n* = 54) and non-NAM acral melanoma (*n* = 78) revealed that, among common melanoma driver genes, mutations in *KIT* and *KRAS* were predominantly found in NAM, whereas those in *BRAF* and *NRAS* occurred almost exclusively in acral melanoma [18]. Regarding the cell cycle pathway, *CDK4*/*CCND1* amplifications were more common in NAM and *CDKN2A/B* loss occurred mostly in acral melanoma [18].

To summarize the reported cases, observed alterations of major genes are as follows: *BRAF*, 10.2% (20/196), range 0–42.9%; *NRAS*, 12.3% (20/162), range 0–30.7%; *KIT*, 18.7% (36/193), range 0–50.0%; and *NF1*, 23.1% (18/78), range 0–50.0% [18,44,46,47,48,49,50,51,52,53,54,55,56,57]. Note that these data on genetic alterations are highly heterogeneous, with different sample sizes, different detection methods, etc.

Common genetic mutations observed in NAM, acral melanoma, and cutaneous melanoma are summarized in Table 1.

## 5. Treatment

Surgical eradication is the mainstay in the treatment for melanoma, including NAM. Postoperative adjuvant systemic therapy can be considered for selected patients at a high risk of metastasis. For metastatic NAM, immune checkpoint inhibitors and *BRAF*-targeted therapy are first-line treatments. Anti-CTLA4 monotherapy or cytotoxic chemotherapy such as dacarbazine have no more been used as first-line. Here, we review the current status of the treatment for NAM.

### 5.1. Surgical Management

#### 5.1.1. Surgery for Primary Tumor

Complete surgical removal of the tumor is the treatment of choice for NAM. However, surgical procedures are sometimes challenging because of the anatomical complexity of the nail unit [23]. Amputation has been performed to achieve tumor eradication since thick NAM sometimes invades the underlying distal phalanx. For cases with no sign of bone invasion, non-amputative digit-preserving surgery has been utilized for in situ or thin NAM [58,59,60,61,62,63]. This technique preserved limb function and improved cosmetic outcomes without decreasing the survival rate in several retrospective studies [58,59,60,61,62,63]. To confirm the safety and usefulness of digit-preserving surgery, a prospective clinical trial is now underway in Japan (JCOG1602, J-NAIL; UMIN000029997). In this trial, eligible patients have invasive NAM with no sign of bone invasion in X-ray radiography. NAM including the periosteum of the distal phalanx is excised, and the defect is closed by skin grafting after confirming clear margins histopathologically.

Lateral surgical margins may be set in accordance with the current guidelines for cutaneous melanoma [64,65]. For instance, the latest National Comprehensive Cancer Network (NCCN) guidelines recommend surgical margins according to the Breslow thickness of the primary melanoma: 5 mm for in situ melanoma, 10 mm for T1 melanoma, 10–20 mm for T2 melanoma, and 20 mm for T3 and T4 melanoma [64]. However, acral melanoma, including NAM, may show a relatively small nodule surrounded by a widely spreading in situ macule. Extensive excision may require amputation at more proximal sites. Narrow-margin excisions have sometimes been attempted at our institute, which yielded no statistically significant difference in survival on multivariate analyses between narrow-margin excision and NCCN-recommended-margin excision, but these results should be confirmed via the prospective collection of data [66].

#### 5.1.2. Sentinel Lymph Node Biopsy

Sentinel lymph node (SLN) biopsy is a standard procedure for staging patients with NAM who have no clinical metastasis [57,67,68,69,70,71,72,73]. Although reported case series involve relatively small sample sizes, a tumor-positive sentinel lymph node is uncommon in thin melanoma. The indication of SLN biopsy specific for NAM is lacking, so we perform SLN biopsy for patients with NAM of ≥T1b thickness in accordance with current guidelines for melanoma. Another group also recommended SLN biopsy to determine the stage and predict the prognosis [57].

#### 5.1.3. Completion Lymph Node Dissection

Based on the two hallmark trials on completion lymph node dissection for SLN-positive patients, MSLT-II and DeCOG-SLT [74,75], close node observation using ultrasound sonography has become the standard management rather than simultaneous completion lymph node dissection. However, only a few patients with NAM may have been included in these trials, and the results should be interpreted with caution. Decision-making on the treatment may need to be made on a case-by-case basis.

For patients with lymphadenopathy in regional nodes, completion lymph node dissection is recommended [64,65]. Elective lymph node dissection beyond the regional nodes may not be necessary [65].

### 5.2. Systemic Adjuvant Therapy for Resected NAM

Adjuvant therapy is offered to patients without evidence of macroscopic metastasis but at a high risk of having microscopic metastasis [64,76]. Several clinical trials have been performed in the adjuvant setting of cutaneous melanoma [77,78,79,80], and adjuvant therapy with nivolumab, pembrolizumab, or dabrafenib/trametinib is commonly used for resected stage IIB/C [79], stage III [78,80,81], and stage IV [81] cutaneous melanoma. However, data on the efficacy of these systemic adjuvant therapies for acral melanoma and NAM are still limited. A retrospective cohort study in China investigating 136 patients with stage III acral melanoma compared the outcome among patients treated with adjuvant anti-PD-1 inhibitor (*n* = 84), adjuvant interferon (*n* = 18) and those without adjuvant therapy (*n* = 34) [82]. They found a lower hazard ratio (0.64; 95% confidence interval, 0.40–1.02) of relapse-free survival in the adjuvant anti-PD-1 inhibitor group than the interferon/observation group, although the difference did not reach statistical significance [82]. Another retrospective study on 90 Chinese patients with stage III cutaneous (*n* = 54) and acral (*n* = 36) melanoma comparing adjuvant anti-PD-1 inhibitor monotherapy and high-dose interferon α2b found that adjuvant anti-PD-1 treatment yielded significantly better recurrence-free survival (hazard ratio, 0.402; 95% confidence interval, 0.183–0.886) and distant metastasis-free survival (hazard ratio, 0.324; 95% confidence interval, 0.122–0.861) in patients with cutaneous melanoma, but a significant difference was not observed in those with acral melanoma [83]. Furthermore, a multicenter study of 78 Japanese patients with melanoma (including 31 acral melanomas) retrospectively analyzed the efficacy and safety profile of adjuvant anti-PD-1 monotherapy and reported that the acral type had a significantly lower 12-month relapse-free survival than other cutaneous types (*p* = 0.029). The acral type was an independent worse prognostic factor on multivariate analysis (*p* = 0.015) [84]. A single-center retrospective study in Japan investigated adjuvant nivolumab (*n* = 5) and other treatments (*n* = 22; 12 patients with interferon β, 4 with chemotherapy, 6 with no treatment) and did not find a superior disease-free survival in the adjuvant nivolumab group over the non-nivolumab group [85]. These results suggest that the adjuvant anti-PD-1 inhibitor monotherapy exert an anti-tumor effect for acral melanoma, but may be less effective than for other types of cutaneous melanoma. Data on the efficacy of adjuvant therapy for NAM are currently unavailable.

### 5.3. Systemic Therapy for Metastatic NAM

Recent advances in immune checkpoint inhibitors and targeted therapy have dramatically changed the management of melanoma and improved patient survival [86,87,88,89,90]. However, it is unclear whether the results of these clinical trials can be applied to patients with NAM, since only a very small number of NAM cases (or none at all) may have been included in these trials (exact data are not available). Although there is a lack of firm evidence of the efficacy of immune checkpoint inhibitors and targeted therapy for NAM, an increasing number of studies on acral melanoma and NAM have recently been reported mainly from Asia, where acral melanomas constitute about half of cutaneous melanoma cases [70,91]. Here, we review these studies and discuss systemic therapy for NAM.

#### 5.3.1. Immune Checkpoint Inhibitors

For unresectable *BRAF* wild-type melanoma, immune checkpoint inhibitors, including anti-PD-1 monotherapy (nivolumab or pembrolizumab) or combination therapy (nivolumab/ipilimumab or nivolumab/relatlimab) are recommended as first-line treatments [64]. Since most NAM cases have wild-type *BRAF*, unresectable cases are principally treated with immune checkpoint inhibitors [52,92,93,94]. Cutaneous melanomas are rich in tumor mutations [45], which is a biomarker for the sensitivity to immune checkpoint inhibitors [95]. However, acral melanoma and NAM have a much lower tumor mutation burden [46], so they are likely to be refractory to immune checkpoint inhibitors [96,97,98]. Indeed, a retrospective study (256 cutaneous, 50 acral, 38 mucosal, and 52 unknown primary melanomas) reported that a complete response to an anti-PD-1 agent was uncommon in acral melanoma (12.0%) compared with the rate in cutaneous melanoma (30.9%) [99]. Other studies focusing on immune checkpoint inhibitors for acral melanoma identified low objective response rates [100,101,102,103,104,105,106,107], although these studies involved relatively small sample sizes of acral melanoma (6–39 patients), and the numbers of NAM cases included are not available. Furthermore, immune checkpoint inhibitors could be less effective for NAM than for acral melanoma. One of the largest retrospective studies from Japan (70 NAMs and 123 non-NAM acral melanomas) reported low objective response rate of anti-PD-1 monotherapy: 21.1% in patients with acral melanoma and 8.6% in patients with NAM [108]. The median overall survival in patients with NAM was significantly worse than for those with non-NAM acral melanoma (12.8 vs. 22.3 months; *p* = 0.03). Another large study with 547 melanomas (including acral melanomas) treated with ipilimumab, however, showed no statistically significant difference of overall survival among melanoma subtypes [109]. A single-arm, open-label, phase II study (CheckMate 172) examined the efficacy and safety profile of nivolumab after the failure of ipilimumab [110]. This study involved a total of 1008 patients, 55 of whom had acral melanoma, and patients with acral melanoma had survival outcomes similar to those of patients with non-acral cutaneous melanoma. There were no meaningful differences in the incidence of grade ≥ 3, treatment-related select adverse events among melanoma subtypes [110]. Taking these findings together, although conflicting results about the efficacy of immune checkpoint inhibitors for acral melanoma and NAM have been reported, these subtypes are potentially refractory to immune checkpoint inhibitors.

In terms of comparisons among immune checkpoint inhibitors for acral melanoma, a systematic review reported the better outcome of the combination therapy of anti-CTLA4 and anti-PD-1 agents (objective response rate: 42.9%) compared with that of each monotherapy [111]. Anti-PD-1 monotherapy showed better objective response rate and progression-free survival than anti-CTLA4 monotherapy (objective response rate 14%–42.9%, progression-free survival 3.2–9.2 months vs. objective response rate 11.4%–25%, progression-free survival 2.1–6.7 months). A recent study involving single-cell RNA sequencing suggested VISTA, ADORA2, TIGIT, and TIM-3 as potential novel immune checkpoints with research value for acral melanoma [112,113].

#### 5.3.2. Targeted Therapy

The latest NCCN guidelines recommend BRAF/MEK inhibitor combination therapy (dabrafenib/trametinib, encorafenib/binimetinib, and vemurafenib/cobimetinib) as one of the first-line treatments for *BRAF*-mutated cutaneous melanoma [64]. As already mentioned, *BRAF* mutations are uncommon in acral melanoma (about 20%) and NAM (about 10%), and BRAF/MEK inhibitor therapy is suitable for only a subset of patients [23,52,92,93,94,114]. Evidence on the efficacy of BRAF/MEK inhibitors for acral melanoma, especially NAM, is accordingly limited. A recent multicenter retrospective study in Japan examined the outcome of BRAF/MEK inhibitors. The objective response rate in acral/mucosal melanoma was relatively high, with no statistically significant difference between acral/mucosal (*n* = 14) and non-acral cutaneous (*n* = 85) melanoma (64.3% vs. 76.5%) [115]. A retrospective study from South Korea reported similar results (objective response rate of 78.9% in 10 acral/mucosal melanomas) [116]. Furthermore, a single-arm phase II trial in China (12 acral, 41 non-acral cutaneous, and 7 unknown primary melanomas) reported the efficacy of dabrafenib/trametinib combination therapy; the 3-year overall survival was 28.8% in the overall population and 35.7% in acral melanoma patients [117]. These results may imply that BRAF/MEK inhibitors can exert efficacy for acral melanoma (including NAM) comparable to that for cutaneous melanoma. Other case series may also support this notion [108,109,110,111,112,113,114,115,116,117,118,119,120,121,122]. However, the long-term efficacy of targeted therapies remains unclear.

Overall, therapy for unresectable NAM is still challenging, even after the clinical application of targeted therapy and immune checkpoint inhibitors, due to the infrequency of *BRAF* mutations and resistance to immunotherapy. Other targeted therapies, including dasatinib, imatinib mesylate, nilotinib, sunitinib, and CDK4/6 inhibitor, have also been applied to acral melanoma [2].

#### 5.3.3. New Classes of Therapeutic Antibodies

Besides naked therapeutic antibodies (anti-PD-1 antibody, etc.), several new classes of antibody drugs, which have been modified to enhance their therapeutic value, have been utilized for solid cancer therapy. These new classes include bispecific antibodies, chimeric antigen receptor T-cell (CAR-T) therapies, and antibody-drug conjugates (ADCs). In this section, we summarize the current knowledge of these novel therapies for melanoma treatment.

In 2021, the results of a randomized controlled study of tebentafusp for the treatment of metastatic uveal melanoma were published [123]. Tebentafusp is a bispecific protein consisting of an affinity-enhanced T-cell receptor fused to an anti-CD3 effector and can redirect T cells to target glycoprotein 100-positive cells (melanoma cells and activated melanocytes). In this trial, a total of 378 patients were randomly assigned to either the tebentafusp group (252 patients) or the control group (126 patients). The control group received the investigator’s choice of therapy with single-agent pembrolizumab, ipilimumab, or dacarbazine. Treatment with tebentafusp resulted in longer overall survival than the control therapy; overall survival at 1 year was 73% in the tebentafusp group and 59% in the control group (hazard ratio for death, 0.51; 95% confidence interval, 0.37 to 0.71; *p* < 0.001) and progression-free survival was 31% in the tebentafusp group and 19% in the control group at 6 months (hazard ratio for disease progression or death, 0.73; 95% confidence interval, 0.58 to 0.94; *p* = 0.01). Treatment-related adverse events were generally tolerable [123]. Based on these results, tebentafusp has been approved by the FDA for the treatment of metastatic uveal melanoma. A clinical trial for its use in metastatic cutaneous melanoma is also now ongoing (NCT02535078). Although limited to patients with HLA-A*02:01, tebentafusp is a potential therapy for metastatic melanoma, including NAM.

CAR-T therapies were first applied to hematological malignancies and have been vigorously expanded to solid tumors [124,125]. T-cell receptors of CAR-T cells are artificially engineered to redirect them to a specific antigen. CAR-T cells kill tumor cells by recognizing target antigens on their surface in a non-MHC-restricted manner [125]. Many preclinical and clinical trials of CAR-T therapy with various potential targets in melanoma have been carried out, with targets including VEGFR2, GD2, cMet, hCD70, gp100, NY-ESO-1, CD20, IL13R-α2, B7H3, and CD19 [123]. All of these trials involved phase I or II non-randomized or single-arm trials, so further investigation is needed [125].

ADCs are another emerging class of therapeutics consisting of a monoclonal antibody linked to a cytotoxic agent through a linker. Upon binding with the cell surface antigen, ADCs are internalized by tumor cells and processed by the endolysosomal system, the linker then being cleaved. The cytotoxic drug is released into the cytoplasm and induces apoptosis of the cell via its cytotoxicity. ADCs have historically been used for hematological malignancies (e.g., ADCs targeting CD30, CD79b, CD33, CD22, CD19, and CD38) but have recently been rapidly expanded to solid tumors [126,127]. FDA-approved ADCs for solid tumors include trastuzumab emtansine and trastuzumab deruxtecan (anti-HER2 ADCs) for breast cancer, enfortumab vedotin (anti-NECTIN-4 ADC) for urothelial cancer, sacituzumab govitecan (anti-TROP-2 ADC) for breast cancer, and tisotumab vedotin (anti-tissue factor ADC) for cervical cancer. Interestingly, NECTIN-4 and TROP-2 are highly expressed in normal skin and its appendages, as well as in various skin cancers [128,129,130,131,132,133,134,135], and anti-NECTIN-4 ADC might be a candidate for unresectable skin cancers, including NAM. Several melanoma-specific ADCs are currently undergoing preclinical and clinical trials. Reported target antigens are gpNMB, PMEL17, HER3, endothelin B receptor, c-KIT, and anexelekto [126,136]. Although no NAM-specific ADCs have been reported, these antigens are likely to be expressed in NAM.

## 6. Conclusions

**Figure 3:**
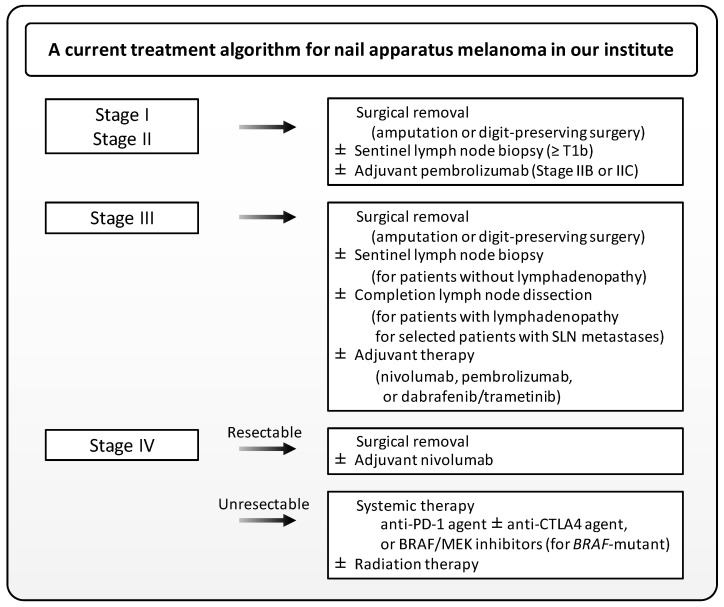
A treatment algorithm for nail apparatus melanoma in our institute. Note that not all FDA-approved drugs are available in Japan. SLN, sentinel lymph node.

This paper has focused on the current management and future perspectives of NAM. Although the diagnosis of NAM at an early stage is sometimes challenging, the diagnosis of advanced NAM is well-established histopathologically. Immunohistochemistry and FISH may provide additional clues to discriminate NAM from benign melanocytic lesions. Dermoscopy can benefit the correct diagnosis of NAM, although standardized treatment guidelines for NAM are lacking. At present, it may be most appropriate to administer treatments in accordance with current guidelines for cutaneous melanoma. A current treatment algorithm for NAM in our institute is given in Figure 3. However, this algorithm should be updated whenever firm evidence comes out, or novel agents are available. Prospective data on the safety profile of digit-preserving surgery will soon become available. Evidence on the efficacy of systemic therapy for unresectable NAM is scarce, but immune checkpoint inhibitors and BRAF/MEK inhibitors have superseded conventional cytotoxic chemotherapy. A lot of novel agents have vigorously been tried for melanoma treatment. Recent genetic analyses have suggested that NAM may differ from cutaneous melanoma and even from acral melanoma. The accumulation of data to better define the optimal management of this uncommon type of melanoma is highly desirable.

## Figures and Tables

**Table 1:** Comparison of common mutations among melanoma subtypes.

Nail Unit Melanoma	Acral Melanoma	Cutaneous Melanoma
*NF1*,	*KIT*,	*BRAF*,
*KIT*,	*NRAS*,	*NRAS*,
*NRAS*,	*BRAF*,	*KIT*,
*BRAF*,	*TERT*,	*NF1*,
*PIK3CA*,	*CDK4*,	*CDKN2A*,
*STK11*,	*NF1*,	*TP53*,
*EGFR*,	*TP53*,	*PTEN*,
*FGFR3*,	*CDKN2A*, etc.	*TERT*, etc.
*PEPN11*, etc.		

## Data Availability

Not applicable.
